# Differential DNA Methylation Analysis without a Reference Genome

**DOI:** 10.1016/j.celrep.2015.11.024

**Published:** 2015-12-08

**Authors:** Johanna Klughammer, Paul Datlinger, Dieter Printz, Nathan C. Sheffield, Matthias Farlik, Johanna Hadler, Gerhard Fritsch, Christoph Bock

**Affiliations:** 1 CeMM Research Center for Molecular Medicine of the Austrian Academy of Sciences, 1090 Vienna, Austria; 2 Children’s Cancer Research Institute, St. Anna Kinderkrebsforschung, 1090 Vienna, Austria; 3 Department of Laboratory Medicine, Medical University of Vienna, 1090 Vienna, Austria; 4 Max Planck Institute for Informatics, 66123 Saarbrücken, Germany

**Keywords:** DNA methylation, differential methylation analysis, bisulfite sequencing, RRBS, reference genome independent analysis, non-model organisms, cross-species comparison, comparative genomics, vertebrate genomes, computational epigenetics

## Abstract

Genome-wide DNA methylation mapping uncovers epigenetic changes associated with animal development, environmental adaptation, and species evolution. To address the lack of high-throughput methods for DNA methylation analysis in non-model organisms, we developed an integrated approach for studying DNA methylation differences independent of a reference genome. Experimentally, our method relies on an optimized 96-well protocol for reduced representation bisulfite sequencing (RRBS), which we have validated in nine species (human, mouse, rat, cow, dog, chicken, carp, sea bass, and zebrafish). Bioinformatically, we developed the RefFreeDMA software to deduce ad hoc genomes directly from RRBS reads and to pinpoint differentially methylated regions between samples or groups of individuals (http://RefFreeDMA.computational-epigenetics.org). The identified regions are interpreted using motif enrichment analysis and/or cross-mapping to annotated genomes. We validated our method by reference-free analysis of cell-type-specific DNA methylation in the blood of human, cow, and carp. In summary, we present a cost-effective method for epigenome analysis in ecology and evolution, which enables epigenome-wide association studies in natural populations and species without a reference genome.

## Highlights

• Bioinformatic method for DNA methylation analysis without a reference genome
• Coverage-optimized high-throughput RRBS protocol validated in nine species
• Antibody-free FACS purification of blood cell types validated in three species
• Analysis of blood cell-type-specific DNA methylation in human, cow, and carp

## Graphical Abstract

**Figure:**
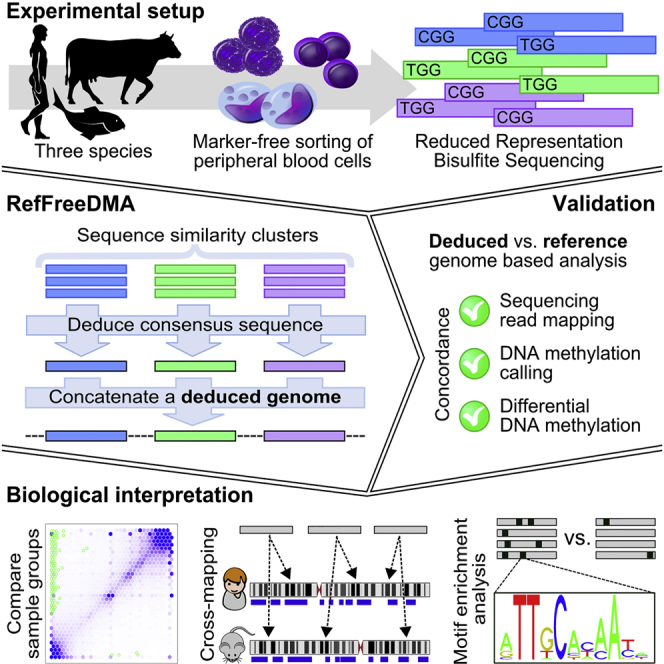

## Teaser

Klughammer et al. describe a method for reference-genome-independent analysis and interpretation of DNA methylation patterns. A combination of experimental and computational advances enables cost-effective DNA methylation analysis in natural populations and species without a reference genome, thus facilitating epigenome-wide association studies in the context of ecology and evolution.

## Background

DNA methylation is an epigenetic mechanism that is indispensable for animal development (Reik, 2007) and also broadly relevant for plant biology (Law and Jacobsen, 2010). Defects in the DNA methylation machinery are associated with widespread changes in cellular identity and interfere with the developmental potential of stem cells (Jones, 2012). Altered DNA methylation patterns are ubiquitous in cancer (Baylin and Jones, 2011, Feinberg and Tycko, 2004), and they have been observed in numerous other diseases (Portela and Esteller, 2010, Robertson, 2005). Moreover, there is mounting evidence for associations between DNA methylation patterns and environmental factors such as stress, nutrition, toxic exposures, and substance abuse (Foley et al., 2009, Mill and Heijmans, 2013).

In humans, epigenome-wide association studies (EWASs) have emerged as a widely used paradigm for linking DNA methylation to environmental exposures and to diseases (Michels et al., 2013, Rakyan et al., 2011). A small number of associations between the epigenome and the environment have also been validated in inbred mouse and rat models, for example, identifying connections between early life exposures and the propensity to subsequently develop certain diseases and behavioral phenotypes. A widely discussed hypothesis posits that epigenetic mechanisms provide a mechanistic link between exposures and diseases, thus contributing to the developmental origins of health and disease in humans (Gillman, 2005, Waterland and Michels, 2007). Furthermore, DNA methylation can be transgenerationally inherited at certain genomic loci (Feil and Fraga, 2011) and may contribute to species evolution (Jablonka and Raz, 2009).

There is tremendous potential in studying environmental influences and epigenetic inheritance not only in laboratory animals, but also in natural populations and non-model organisms. For example, animals in the wild are often exposed to complex evolutionary pressures and ecological interactions that cannot be modeled in the laboratory. Initial studies along these lines have suggested a role of epigenetics in the evolution of Darwin’s finches (Skinner et al., 2014) and in speciation among marsupials (O’Neill et al., 1998), and they identified DNA methylation as a potential source of random variation in natural populations of fish (Massicotte et al., 2011) and songbirds (Liebl et al., 2013, Schrey et al., 2012).

However, systematic epigenetic studies in natural populations and non-model organisms have been hampered by the lack of methods for high-resolution and high-throughput DNA methylation analysis that work well across a broad range of species. To date, most studies of DNA methylation in ecology and evolution have relied on low-throughput, gel-based assays such as MS-AFLP (Schrey et al., 2013). Much more powerful assays are being used for DNA methylation analysis in human, including the Infinium microarray, whole-genome bisulfite sequencing (WGBS), and reduced representation bisulfite sequencing (RRBS). However, none of these assays is directly applicable for studying DNA methylation in natural populations and non-model organisms: The Infinium assay requires a commercial microarray that is only available for the human genome (Bibikova et al., 2011); WGBS is excessively expensive when studying more than a handful of samples (Beck, 2010), and RRBS suffers from the technical complexity of the original protocol (Gu et al., 2011) and from concerns that the restriction enzyme MspI may not provide good genome coverage in other species. Furthermore, there is a general lack of bioinformatic methods for analyzing sequencing-based DNA methylation data in the absence of a high-quality reference genome and in genetically diverse populations for which existing reference genomes would unduly bias the analysis.

**Figure 1:**
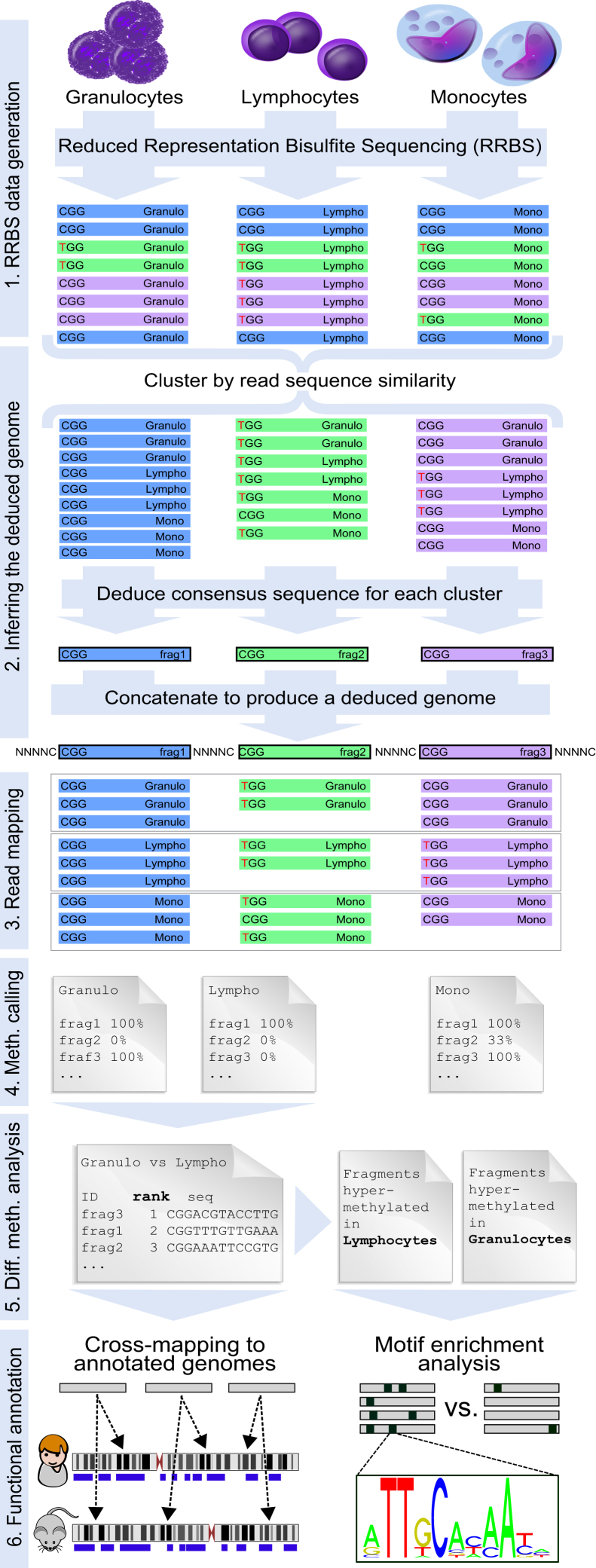
DNA Methylation Analysis without a Reference Genome Workflow for reference-genome-independent analysis of differential DNA methylation using an optimized RRBS protocol and the RefFreeDMA software. Colored bars represent RRBS sequencing reads, and identical colors indicate high sequence similarity. Bisulfite-converted MspI restriction sites are shown at the beginning of each read (CGG for methylated sites and TGG for unmethylated sites). To derive a deduced genome, reads from all samples are clustered by sequence similarity, and a consensus sequence is determined. These deduced genome fragments (black-edged bars) are concatenated into one deduced genome, to which the RRBS reads for each sample are mapped. DNA methylation levels are obtained by counting the number of Cs versus Ts for individual cytosines in the deduced genome (this step typically focuses on CpG sites, but the method also supports the analysis of non-CpG methylation). Differential methylation analysis is performed by comparing site-specific and fragment-specific DNA methylation levels between sample groups. Finally, the identified differentially methylated fragments are analyzed by cross-mapping to well-annotated genomes of other species (e.g., mouse or human) and by motif enrichment analysis (e.g., for identifying enriched transcription factor binding sites).

Here, we describe an integrated approach for analyzing DNA methylation at single-base-pair resolution in a broad range of species. We combine an optimized high-throughput RRBS protocol with a tailored computational method called RefFreeDMA in order to detect differential DNA methylation without a reference genome. RefFreeDMA constructs a deduced genome directly from RRBS sequencing reads, it maps the sequencing reads to the deduced genome, performs DNA methylation calling, and identifies differentially methylated cytosines and DNA fragments (Figure 1). We validated our method by studying blood cell-type-specific DNA methylation in three species (human, cow, and carp), benchmarking the reference-free analysis against a reference-based analysis using the existing reference genomes. The experimental protocol was also validated in six additional vertebrate species (rat, mouse, dog, chicken, sea bass, and zebrafish). We expect that the described method will be broadly useful for DNA methylation analysis in non-model organisms, for example, to identify and interpret DNA methylation differences between samples (e.g., different cell types) or groups of individuals (e.g., animals that have been exposed to different environments).

## Results

### High-Throughput DNA Methylation Mapping in Diverse Animal Species Using RRBS

**Figure 2:**
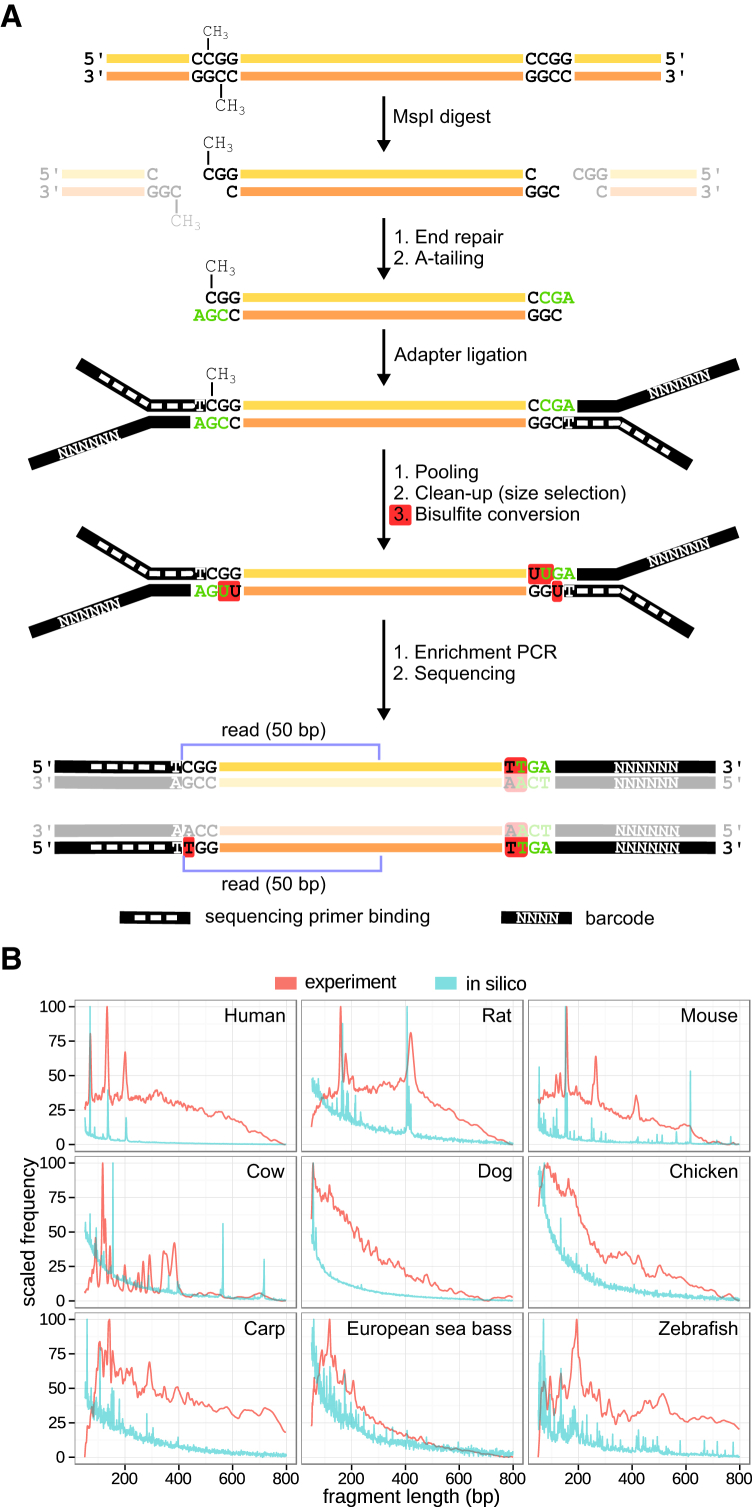
An Optimized RRBS Protocol Validated in Nine Species (A) Schematic outline of RRBS library preparation and the corresponding sequencing reads. (B) Computationally predicted (blue) and experimentally measured (red) fragment length distribution of RRBS libraries in nine vertebrate species. Predictions were based on in silico MspI restriction digests of the reference genomes using the BSgenome R package. Experimental results were obtained by electrophoresis (Experion DNA 1k chip). In species with a reference genome, concordance between predicted and experimentally measured peaks can be used to confirm successful RRBS library preparation.

RRBS enables genome-scale DNA methylation mapping at single-base-pair resolution for a fraction of the cost of WGBS (Meissner et al., 2005). It exploits the highly characteristic distribution of DNA methylation in vertebrate genomes, which occurs mainly at CpG dinucleotides. DNA is digested with the restriction enzymes MspI (restriction site: C^∧^CGG) and/or TaqI (restriction site: T^∧^CGA), which are insensitive to DNA methylation at the central CpG, and short size-selected restriction fragments are subjected to bisulfite sequencing (Figure 2A).

We adapted an existing RRBS protocol (Boyle et al., 2012) and optimized it for genome coverage and sample throughput (see Experimental Procedures for details). The optimized protocol increases the number of covered CpG sites from ∼2.5M to ∼4M (human genome, using the MspI enzyme), and it allows a single person to process up to 192 samples per week. For most vertebrates, good sequencing coverage can be obtained when 6–12 barcoded samples are sequenced on a single lane of Illumina HiSeq, which makes the protocol approximately 10-fold cheaper than WGBS. To validate the assay, we generated RRBS libraries for nine species (human, rat, mouse, cow, dog, chicken, carp, sea bass, and zebrafish). These libraries showed characteristic fragment length distributions, which reflect the distribution of CpG-rich repetitive elements in these species and which provide a convenient metric for assessing the quality of RRBS libraries prior to sequencing (Figure 2B).

**Figure 3:**
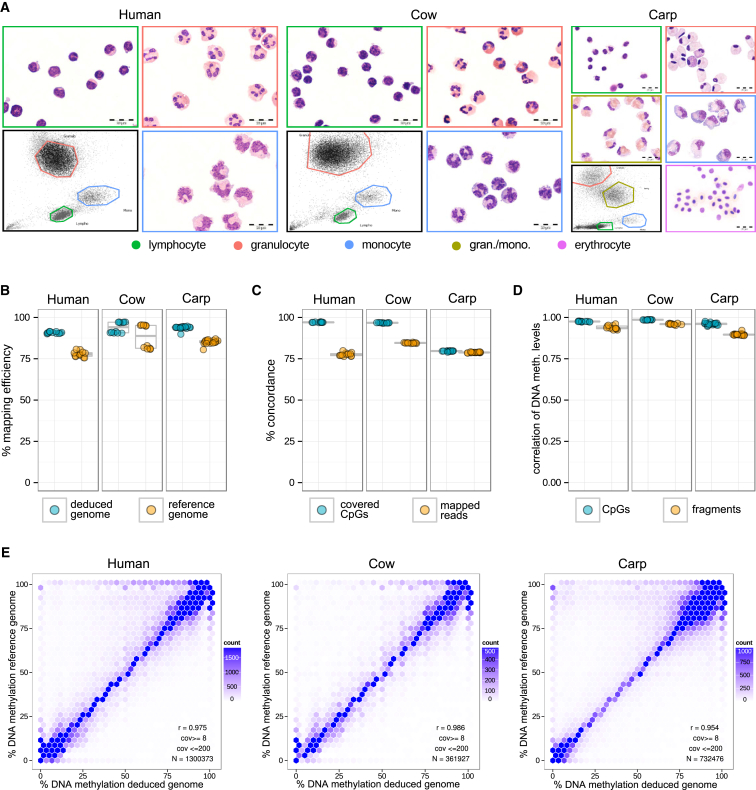
Validation of Reference-Free DNA Methylation Mapping (A) Representative images (Giemsa-stained cytospins at 100× magnification) of blood cell populations that were purified by FACS using an antibody-independent protocol based on forward scatter (x axis) and side scatter (y axis). Gated cell populations are highlighted in different colors, and their DNA was used for RRBS library preparation. (B) Percent mapping efficiency (alignment rate) for RRBS reads using the deduced genome versus the reference genome. Mapping rates are expectedly lower than 100% for the reference-free method because low-confidence reads are used during alignment but not for building the deduced genome. (C) Percentage of CpGs and sequencing reads with concordant mapping between the two approaches in non-repetitive genomic regions (see Figure S3A for details). (D) Pearson correlation of DNA methylation levels for the two approaches, compared at the level of CpG sites and deduced genome fragments using RefFreeDMA’s standard filtering criteria (coverage of least eight and not more than 200 mapped reads). (E) DNA methylation scatterplots at the level of CpG sites (r, Pearson correlation; N, number of CpGs; cov, minimum and maximum read coverage used for filtering).

Using our optimized RRBS protocol, we established a DNA methylation dataset for the major nucleated cell populations in peripheral blood of three species (human, cow, and carp), with four biological replicates per cell type and species. The human and cow datasets comprise granulocytes, monocytes, and lymphocytes, whereas the carp dataset also includes nucleated erythrocytes and one additional leukocyte population that morphologically resembles granulocytes and monocytes (Figure 3A). In total, the dataset comprises 44 blood cell samples from three species and 789 million sequencing reads (Table S1). All cell types were fluorescence-activated cell sorting (FACS) purified based on forward and side scatter alone, demonstrating the feasibility of separating blood cell types in species that lack suitable FACS antibodies. The purity of the sorted cell populations was assessed visually through cytospins, and it exceeded 95% in all samples. Here, our analysis focuses on DNA methylation differences between these cell populations, but the same sorting strategy can also be used for minimizing the impact of differences in cell composition between individuals, which is a major confounder in human EWAS (Houseman et al., 2012, Jaffe and Irizarry, 2014).

### RefFreeDMA: Analyzing Differential DNA Methylation without a Reference Genome

We devised a workflow for reference-free DNA methylation analysis consisting of six main steps (Figure 1): (1) preparation and sequencing of RRBS libraries, (2) inference of a deduced genome from the RRBS sequencing reads, (3) read alignment to the deduced genome, (4) DNA methylation calling, (5) identification and ranking of differentially methylated CpGs and deduced genome fragments, and (6) functional annotation of differential DNA methylation. RefFreeDMA is implemented as a Linux-based software pipeline, supporting small to moderately sized analyses on a desktop computer (e.g., 40-hr total runtime for 20 samples), whereas large analyses are efficiently parallelized on a computing cluster. A detailed overview of the RefFreeDMA pipeline is provided as a Unified Modeling Language (UML) diagram in Figure S1.

A key aspect of RefFreeDMA is the construction of a deduced genome directly from the RRBS reads. This deduced genome is not based on classical de novo assembly of bisulfite sequencing reads, which is computationally expensive and would require very deep sequencing. Rather, we exploit a specific characteristic of RRBS with its defined fragment start and end positions at MspI restriction sites to simplify the problem. RefFreeDMA constructs the deduced genome by clustering the RRBS reads from all samples in a given species according to their sequence similarity, followed by inference of the consensus sequence for each read cluster. In the consensus sequence, positions with both cytosines (Cs) and thymines (Ts) among the clustered reads are retained as Cs (Figure 1), given that they are likely to reflect genomic cytosines that are methylated and protected from bisulfite sequencing in some but not all samples. We developed an efficient two-step approach in which all quality-filtered, non-duplicate sequencing reads are initially clustered in an approximate and computationally efficient manner, followed by a more precise and computationally demanding finalization step (see Experimental Procedures for details). Finally, all consensus sequences are concatenated with spacer sequences (i.e., stretches of Ns) to facilitate computational processing, resulting in a deduced genome that is specific for a given species and analysis but shared among all samples contributing to the analysis.

The subsequent steps of read alignment, DNA methylation calling, and differential methylation analysis are performed in much the same way as for DNA methylation analysis with a reference genome (Bock, 2012). Specifically, we use BSMAP/RRBSMAP (Xi et al., 2012, Xi and Li, 2009) for read alignment and a custom DNA methylation calling script (Bock et al., 2010) for calculating the fraction of methylated reads at each CpG position in the deduced genome. Differentially methylated CpGs and deduced genome fragments between sample groups are then identified using a modified t test statistic as described for the RnBeads software (Assenov et al., 2014). The analysis gives rise to lists with individual CpGs as well as deduced genome fragments ranked by their degree of differential methylation.

In a final step, the top-ranking differentially methylated fragments are exported as FASTA/FASTQ files, which provide the basis for biological interpretation by cross-mapping to well-annotated genomes and by reference-free motif enrichment analysis. The principle behind cross-mapping is to link deduced genome fragments in the analyzed species to orthologous regions in well-annotated genomes of other vertebrate species and to use the genome annotations that are available in the latter species (e.g., genes, transcription factor binding sites, histone modifications, and DNase hypersensitivity sites) for cross-species enrichment analysis. This approach is of course limited to genomic regions that are conserved across species; hence, it is most powerful for species that are closely related to well-characterized model organisms.

Motif enrichment analysis provides an alternative approach to biological interpretation that is independent of any reference genomes. It is based on the observations that transcription factor binding motifs are highly conserved across all vertebrates (Nitta et al., 2015) and that DNA methylation levels at motif sequences have been shown to correlate with cell-type-specific transcription factor binding (Bock et al., 2012, Feldmann et al., 2013, Stadler et al., 2011). By analyzing motif enrichment among differentially methylated DNA fragments using existing databases (such as JASPAR; Mathelier et al., 2014) and software tools (such as AME; McLeay and Bailey, 2010), it is possible to gain insight into the regulatory mechanisms that distinguish the studied cell types and sample groups.

### Validating Reference-Free DNA Methylation Analysis across Three Species and 44 Samples

To validate our approach, we performed reference-free analysis of the RRBS blood cell dataset (Figure 3A) and compared the results to those obtained by reference-based analysis of the same data (see Experimental Procedures for details). The fraction of aligned reads was in the range of 90% to 98% for the deduced genomes and slightly lower (75% to 95%) for the published reference genome of each species (Figure 3B; Table S1). The number of covered CpGs was predominantly species specific (3–4 million for human, ∼3 million for cow, and 1.5–2 million for carp) and broadly similar between the reference-based and reference-free analysis. Average DNA methylation levels at CpG sites were also similar for both approaches, whereas the observed C-to-T conversion rates at non-CpG sites were substantially lower in the reference-free analysis (Table S1). This is because ubiquitously unmethylated Cs—which in vertebrates are mostly found in non-CpG context—are counted as Ts by the reference-free analysis (case 4 in Figure S2) and therefore do not contribute to high non-CpG conversion rates. To circumvent this potential problem our RRBS protocol uses methylated and unmethylated spike-in controls to monitor bisulfite conversion rates (Table S1), rather than relying on non-CpG conversion rates. The issue can also be avoided altogether by sequencing a single RRBS sample without bisulfite conversion and including it in the analysis. Finally, to assess the comparative performance of our reference-free method, we benchmarked it against simply cross-mapping the RRBS reads for carp to the well-annotated genomes of human, mouse, and zebrafish. The results showed a one to two orders of magnitude higher genome-wide CpG coverage using RefFreeDMA than observed for the basic cross-mapping approach (Table S2).

We also compared the alignment of individual reads, the coverage of individual CpGs, and the DNA methylation levels of single CpGs and deduced genome fragments between the two approaches. To that end, the deduced genome fragments were aligned to the corresponding reference genome, allowing us to link most RRBS fragments (human: 1,254,324 out of 1,522,786; cow: 1,276,537 out of 1,521,946; and carp: 455,821 out of 780,757) to their putative position in the reference genome. More than 75% of reads and CpGs in non-repetitive regions where concordantly mapped by both approaches (Figure 3C), whereas the agreement was much lower for repetitive regions and reads that map to multiple positions in the genome (Figure S3A). We investigated these discrepancies and identified four scenarios in which there may be deviations between the reference-free method and the reference-based method (Figure S2). Most frequently, a sequencing read maps to multiple positions throughout the reference genome, and the aligner randomly assigns it to one of these positions. We indeed observed similarly low concordance rates in repetitive regions when running the reference-based method twice with different random seed parameters (Figure S3A). Based on these results, it might even be argued that the clustering and combining of highly similar repetitive reads into a single consensus provide a more appropriate way of handling multimapping reads than their random assignment in the reference-based analysis, and similar approaches have successfully been used for studying epigenetic marks in repetitive regions of the genome (Bock et al., 2010, Day et al., 2010). Finally, despite these special cases, we observed excellent agreement between the two approaches when plotting alignment positions across a representative chromosome (Figure S3B), and the DNA methylation values obtained with the two approaches were highly correlated in all samples and all species—with Pearson correlation coefficients above 0.9 across all CpGs and fragments and above 0.95 for those CpGs and fragments that have good sequencing coverage (Figures 3D, 3E, and S3C).

### Reference-Free Analysis of Differential DNA Methylation between Cell Types of the Blood

**Figure 4:**
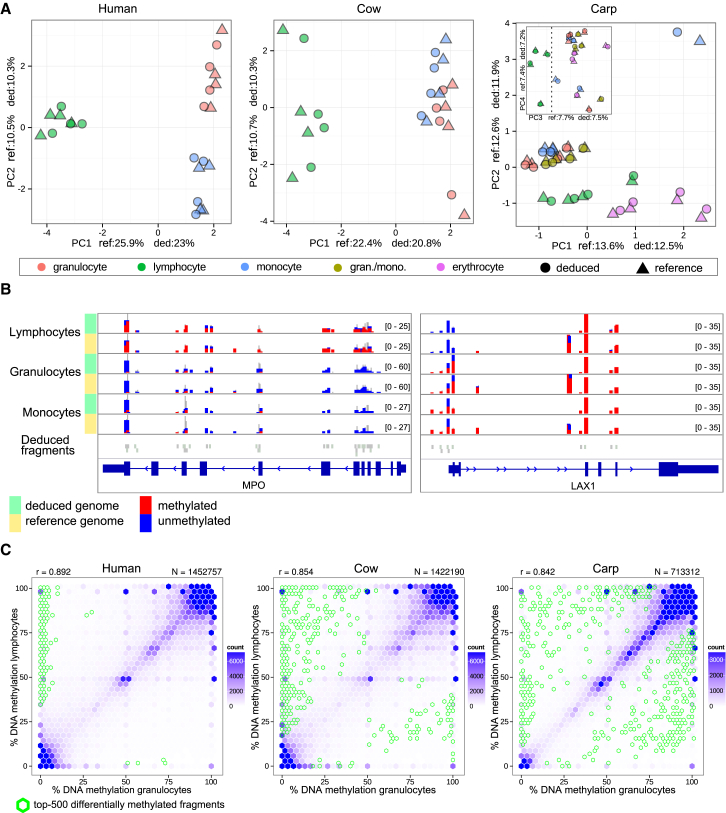
Differential DNA Methylation Analysis without a Reference Genome (A) Global concordance between reference-free and reference-based DNA methylation analysis illustrated by principal component analysis. Shown are the first two principal components (x axis and y axis) for the reference-free (circles) and reference-based (triangles) approaches as well as the percentage of variance explained by these principal components. The inset for carp shows the third and fourth principal components, which provides clearer separation of lymphoid versus myeloid cell types. (B) Representative genome browser tracks displaying DNA methylation levels at single CpG sites as determined by the reference-free and reference-based approach, focusing on genes with known myeloid (MPO) and lymphoid (LAX1) function. The “Deduced fragments” track depicts the mapping between deduced genome fragments (gray boxes) and the reference genome. (C) DNA methylation scatterplots showing differential DNA methylation in granulocytes (x axis) versus lymphocytes (y axis) based on the reference-free approach. Means across four biological replicates per cell type are shown, and the green hexagons indicate the top-500 most differentially methylated fragments (r, Pearson correlation; N, number of deduced genome fragments). Matched scatterplots for the reference-based analysis are shown in Figure S4D.

Importantly, the reference-free method was able to recapitulate the known biological similarities and differences among the different blood cell types in almost perfect concordance with the reference-based method (Figure 4A). Many genes with a known role in hematopoietic cells were identified by both methods, as illustrated by the myeloid-specific MPO gene and the lymphoid-specific LAX1 gene (Figure 4B). There was also strong correlation (r ≥ 0.95) between the differential DNA methylation ranks obtained with the two methods in all three species (Figure S4A). Furthermore, the vast majority of the top-1,000 differentially methylated fragments identified by the reference-free method were also among the top-1,000 or top-5,000 differentially methylated regions based on the reference-based method (Figure S4B). The magnitude of the DNA methylation differences calculated by either method were also highly correlated (Figure S4C). Furthermore, both methods identified a consistent and biologically interesting trend toward increased DNA methylation levels in lymphoid as opposed to myeloid cells, which was very prominent in human, weaker in cow, and essentially absent in carp (Figures 4C and S4D), suggesting species-specific differences in the genome-wide regulation of DNA methylation in the hematopoietic system.

**Figure 5:**
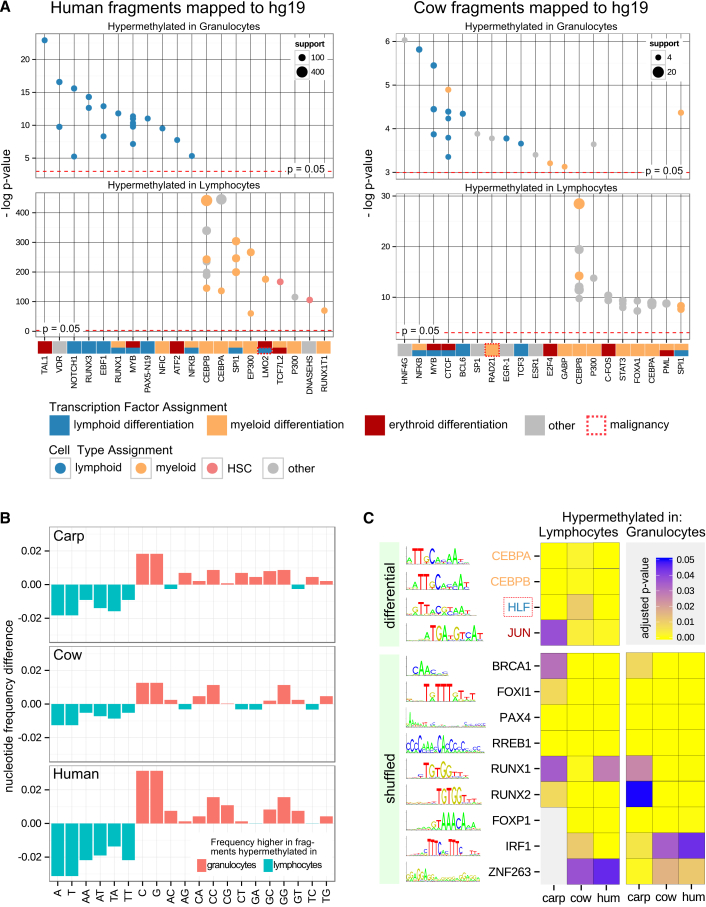
Biological Interpretation of DNA Methylation Differences (A) Region enrichment analysis for differentially methylated deduced genome fragments that have been cross-mapped to the human genome (hg19). The top-20 enriched region sets obtained by LOLA analysis are shown. Uncorrected p values are plotted on the y axis, and the number of overlapping regions is indicated by bubble size. Each dot represents a region set in the database, and the red dashed line indicates p values of 0.05. Similar plots for carp and for cross-mapping to the mouse genome (mm10) are shown in Figure S5B. Cell-type-specific gene functions are based on literature search and indicated through colored boxes on the x axis. (B) Nucleotide frequency differences between the top-500 deduced genome fragments with increased DNA methylation in granulocytes versus lymphocytes (red) and vice versa (blue). (C) Enrichment of known sequence motifs associated with transcription factor binding sites among the top-500 deduced genome fragments with increased DNA methylation in granulocytes versus lymphocytes (right) and vice versa (left). The motif analysis used either the opposing group (“differential”) or randomly shuffled sequences with the same mono- and dinucleotide composition (“shuffled”) as background. The diagram only shows motifs that were enriched in all three species; the complete sets of enriched transcription factor binding motifs are shown in Figures S5D and S5E.

We pursued two complementary approaches for interpreting the identified DNA methylation differences without a reference genome for the target species. First, we cross-mapped the deduced genome fragments obtained in each species to the human and mouse genome, for which extensive functional genomics data exist from projects such as ENCODE (ENCODE Project Consortium, 2004), IHEC (http://www.ihec-epigenomes.org/), and BLUEPRINT (Adams et al., 2012). Cross-species mapping rates were expectedly low, amounting to ∼20% for human and cow and ∼10% for carp at a maximum mismatch rate of 20%. (Figure S5A). Nevertheless, for those deduced reference fragments that did map, we were able to perform enrichment analysis relative to the extensive biological annotations of the human and mouse genomes. Fragments that were less methylated in lymphocytes as compared with granulocytes (hypermethylated in granulocytes) were often associated with lymphoid-specific regulatory elements and transcription factor binding mapped by ChIP-seq and similar technologies (Figures 5A and S5B). The enrichment was not always consistent between species, but we found recurrent and biologically meaningful associations. Most notably, the binding sites of two key myeloid transcription factors, CEBPA and CEBPB (Akagi et al., 2010, Rosenbauer and Tenen, 2007), were hypermethylated in both human and cow lymphocytes, and binding sites of MYB, a transcription factor implicated in lymphocyte and erythrocyte development (Greig et al., 2008), were hypermethylated in human and cow granulocytes. In contrast, carp appears to be too evolutionary distant to obtain interesting results by cross-mapping to mammalian genomes (Figure S5B).

Second, we exploited the fact that transcription factor binding motifs are much more conserved than most regulatory elements (Nitta et al., 2015) and performed alignment-free motif enrichment analysis for those deduced reference fragments that were most differentially methylated between lymphocytes and granulocytes. In all three species, there was a higher ratio of GC-rich and CpG-rich motifs among fragments that are hypermethylated in granulocytes (Figures 5B and S5C), which we corrected for in the motif analysis by using random sequences with matched base composition as controls (see Experimental Procedures for details). Those fragments that were less methylated in lymphocytes (hypermethylated in granulocytes) were enriched for 29 sequence motifs, of which four were shared across two species (EGR2, KLF5, KLF1, and RREB1; shown in Figure S5D). Those fragments that were less methylated in granulocytes (hypermethylated in lymphocytes) were enriched for 40 sequence motifs, and four motifs were shared between all three species (CEBPA, CEBPB, HLF, and JUN) (Figures 5C and S5D). Three of these transcription factors are well-established regulators of myeloid cell differentiation (Akagi et al., 2010, Orkin, 1995, Rosenbauer and Tenen, 2007), whereas HLF is associated with hematopoietic stem cells (Gazit et al., 2013). Finally, we also searched for motifs that were enriched in lymphocyte-specific as well as in granulocyte-specific differentially methylated fragments (Figures 5C and S5E), and a total of 27 sequence motifs were identified, of which six were shared across all three species (BRCA1, FOXL1, PAX4, RREB1, RUNX1, and RUNX2). Of these, RUNX1 and RUNX2 in particular are known to play a role in both lymphoid and myeloid cell differentiation and function (Klunker et al., 2009, Liebermann and Hoffman, 2002, Tenen et al., 1997).

## Discussion

We present an integrated experimental and computational method for DNA methylation analysis and interpretation in non-model organisms, unsequenced species, and natural populations. Our method addresses a major bottleneck for epigenome studies in the context of comparative genomics, ecology, and evolution, where whole genome bisulfite sequencing is rarely affordable for sufficiently large cohorts and other widely used methods such as MS-AFLP are strongly limited in the information they can provide.

On the experimental side, our method uses an optimized 96-well RRBS protocol, which provides an excellent trade-off between single-base-pair resolution, affordable cost, and practical feasibility for studies with hundreds (or even thousands) of individuals. Building upon the track record of RRBS in mouse and human and the popularity of reduced representation genome sequencing assays such as RAD-seq (Baird et al., 2008) and GBS (Elshire et al., 2011) for research in natural populations and non-model organisms, we expect our method to be broadly useful for EWASs in the context of ecology and evolution.

The described method should be applicable to any animal and plant species with appreciable levels of DNA methylation, and it is readily adapted to different genome compositions and sequencing depths by selecting an appropriate restriction enzyme (or enzyme combinations). Here we focused on vertebrates, where DNA methylation is largely restricted to CpG dinucleotides and the MspI restriction enzyme is an ideal choice. MspI enriches for CpG islands and gene promoters, while also providing a broad sampling of other genomic regions such as enhancers, gene bodies, CpG island shores, and repetitive elements. Furthermore, every read contains at least one CpG (at the MspI restriction site), which increases cost-effectiveness for vertebrate genomes. Importantly, our method can be used to map not only CpG methylation, as we demonstrate here, but also non-CpG methylation (Ziller et al., 2011), which is widespread among non-vertebrate species and also present in certain vertebrate cell types.

On the computational side, we developed the RefFreeDMA method and software to build a deduced genome directly from the bisulfite sequencing reads, to quantify DNA methylation at the level of single CpG sites and deduced fragments, and to detect and rank DNA methylation differences between samples and sample groups. RefFreeDMA overcomes relevant limitations of an existing method that uses de novo assembly of MeDIP-seq reads (Kaspi et al., 2014), namely low resolution, susceptibility to biases, and lack of quantification, and it is more powerful and more widely applicable than read mapping to the genome of a related species (Weyrich et al., 2014), which requires a closely matched genome and a second, unconverted library. Furthermore, we present two approaches (cross-mapping and motif enrichment analysis) for interpreting the identified differentially methylated regions in the absence of a reference genome.

To validate our method, we established and analyzed a cross-species DNA methylation dataset comprising multiple blood cell types in two mammalian species (human and cow) and one fish (carp). All cell types were enriched to >95% purity by a sorting strategy that is particularly useful for working with non-model organisms because it does not require any species-specific antibodies. Bioinformatic analysis in the three species with and without the respective reference genomes gave rise to consistent and informative results. For example, we observed that the most differentially methylated fragments in the two mammalian species were predominantly hypermethylated in lymphocytes, whereas no such bias was present in carp (Figures 4C and S4D). We also identified characteristic binding motifs of lineage-specific transcription factors that were consistently enriched among differentially methylated fragments of all three species (Figure 5C).

Despite the good results that we obtained in our validation of RefFreeDMA, there are several inherent limitations of reference-free DNA methylation analysis that potential users of our method should keep in mind. First, repetitive elements with high sequence similarity can get merged into a single deduced genome fragment, which is why RefFreeDMA tends to report moderately fewer covered CpGs than we obtained using reference-based analysis. Second, cytosines that are unmethylated in all samples of one species will not be represented in the deduced genome (case 4 in Figure S2), unless one RRBS sample is sequenced without bisulfite conversion and added to the analysis. Third, our method does not perform de novo assembly of deduced genome fragments, which would require substantially deeper and broader sequencing coverage than is typically affordable. It can therefore happen that the same CpG is included twice in two partially overlapping fragments (case 2 in Figure S2). However, based on our analysis of the validation dataset, this type of bias appears to be negligible (Figure S4C).

In summary, we expect that RefFreeDMA in combination with our optimized RRBS protocol will be useful for researchers who are interested in analyzing DNA methylation in non-model organisms without the need of a reference genome. Apart from assessing cell-type-specific DNA methylation as demonstrated here, other applications of RefFreeDMA may include EWASs for phenotypic differences in natural populations, agricultural research on the epigenetic effect of different feeds, drugs, and rearing conditions, and meta-epigenome studies of DNA methylation in entire ecosystems.

## Experimental Procedures

### Sample Acquisition

For human, cow, and carp, 5–10 ml of peripheral blood was obtained from two male and two female individuals, anti-coagulated by 2 mg/ml K_2_EDTA and processed within 1 hr after collection. Human blood samples were obtained by venipuncture from healthy donors by a qualified physician. All donors provided informed consent. The study was conducted in accordance with the principles laid down in the Declaration of Helsinki, overseen by the ethics commission of the Medical University of Vienna. Cow blood samples were obtained post-mortem from a slaughterhouse. Carp blood samples were obtained post-mortem from a fish vendor. For the other species (mouse, rat, dog, chicken, sea bass, and zebrafish), purified DNA was provided by the collaborators listed in the Acknowledgments.

### Cell Purification

Leukocytes were isolated from whole blood by removing the erythrocytes through hypotonic lysis. Specifically, 5 ml of whole blood was incubated with 9 ml ddH_2_O for 1 min. The lysis was stopped by adding 1 ml of 10× PBS to the sample. Leukocytes were pelleted by centrifuging for 5 min at 550 *g*. If the pellet was still red, a second round of lysis was initiated by resuspending the pellet in 1 ml 1× PBS. Subsequently, 4.5 ml of ddH_2_O was added and after 30 s the lysis reaction was stopped by adding 0.5 ml 10× PBS. Leukocytes were pelleted by centrifuging for 3 min at 550 *g*. Finally, the pellet was washed in 1 ml 1× PBS and then resuspended in 500–800 μl RPMI-1640 medium supplemented with 10% fetal calf serum (FCS). The cell suspension was then filtered into a FACS tube, and cell populations were sorted by FACS based on their forward and side scatter properties. Sorting was performed on a BD FACS Aria 1 with a 70-μm nozzle, which allowed for a maximum sorting speed of 30,000 events per second. For each population, between 500,000 and 3 million cells were obtained. Giemsa stained cytospins were produced for each sorted cell population, and the purity was assessed at 100× magnification.

### DNA Isolation

The Allprep DNA/RNA Mini kit (QIAGEN) was used for DNA isolation. Cells were lysed in 600 μl Buffer RLT Plus supplemented with 1% β-Mercaptoethanol and vortexed thoroughly for at least 5 min. The procedure of isolating DNA and RNA was performed according to protocol. DNA was stored at −20°C.

### RRBS Library Preparation

For RRBS, 100 ng of genomic DNA was digested for 12 hr at 37°C with 20 units of MspI (New England Biolabs, R0106L) in 30 μl of 1× NEB buffer 2. To retain even the smallest fragments and to minimize the loss of material, end preparation and adaptor ligation were performed in a single-tube setup. End fill-in and A-tailing were performed by addition of Klenow Fragment 3′ > 5′ exo- (New England Biolabs, M0212L) and dNTP mix (10 mM dATP, 1 mM dCTP, 1 mM dGTP). After ligation to methylated Illumina TruSeq LT v2 adaptors using Quick Ligase (New England Biolabs, M2200L), the libraries were size selected by performing a 0.75× cleanup with AMPure XP beads (Beckman Coulter, A63881). The libraries were pooled in combinations of six based on qPCR data and subjected to bisulfite conversion using the EZ DNA Methylation Direct Kit (Zymo Research, D5020) with the following changes to the manufacturer’s protocol: conversion reagent was used at 0.9× concentration, incubation performed for 20 cycles of 1 min at 95°C, 10 min at 60°C, and the desulphonation time was extended to 30 min. These changes increase the number of CpG dinucleotides covered by reducing double-strand break formation in larger library fragments. Bisulfite-converted libraries were enriched using PfuTurbo Cx Hotstart DNA Polymerase (Agilent, 600412). The minimum number of enrichment cycles was estimated by qPCR. After a 2× AMPure XP cleanup, quality control was performed using the Qubit dsDNA HS (Life Technologies, Q32854) and Experion DNA 1k assays (BioRad, 700-7107). RRBS libraries were sequenced on the Illumina HiSeq 2000 platform in 50-bp single-read mode.

### Bisulfite Conversion Controls

In order to monitor the efficiency of the bisulfite conversion and to check for underconversion of unmethylated cytosines as well as overconversion of methylated cytosines, custom-designed and synthesized methylated and unmethylated oligonucleotides were spiked into each sample at a concentration of 0.1% of the genomic DNA. For each sample, sequencing reads were aligned to the control sequences using Bismark with default settings (Krueger and Andrews, 2011). Conversion metrics are reported in Table S1.

### RRBS Data Preprocessing

Sequencing data were processed with illumina2bam-tools v.1.12, and the resulting BAM files were converted to fastq format using SamToFastq.jar (picard-tools v.1.100) with the INCLUDE_NON_PF_READS parameter set to FALSE. All reads were trimmed for adaptor sequences and low-quality sequences using trimgalore v.0.3.3 (http://www.bioinformatics.babraham.ac.uk/projects/trim_galore/) with the following command: *trim_galore -q 20–phred33 -a “AGATCGGAAGAGCACACGTCTGAACTCCAGTCAC”–stringency 1 -e 0.1–length 16–output_dir $output_dir $input_fastq.*

### Derivation of a Deduced Genome

Based on the trimmed RRBS reads for a given species and analysis, a deduced genome is constructed in six steps: (1) *Pre-filtering.* To reduce the number of reads that need to be processed, one representative read is kept for each read sequence and sample. Furthermore, reads that stand a high chance of arising from sequencing errors are discarded by requiring that each read occurs at least twice among four samples after converting all Cs to Ts. (2) *Preliminary read grouping.* To be computationally effective, we perform read grouping initially by exact string matching. Reads that share the same sequence in their fully converted form (all Cs replaced by Ts) are combined into one pre-consensus sequence by assigning a C to each position at which at least 5% of the reads contain a C in their unconverted form. (3) *Consensus building.* To combine highly similar but not identical fragments into one consensus, the pre-consensus fragments are grouped by sequence similarity using an all-against-all alignment of the C to T converted fragments with Bowtie2 v.2.2.3 (Langmead and Salzberg, 2012) using the following command: *bowtie2 -t -q–phred33–end-to-end -N 1 -L 22–norc–n-ceil “L,0,0.2”–mp 3–np 0–score-min “L,-0.6,-0.6” -k 300 -D 3–rdg “20,20”–rfg “20,20” -p 4 -x $reference -U $fastq -S $out_sam*. Fragments that match with less than 8% maximum mismatch ratio are merged by assigning them to the largest available group. For each group, a consensus sequence is deduced by assigning the majority base to each position, while assigning Cs to all positions at which at least 5% of the fragments contain a C. (4) *Consensus refinement.* For those groups in which some fragments exhibit more than 5% mismatches relative to the consensus, the diverging reads are assigned to separate groups, and a new consensus is built for the respective groups. This procedure is repeated until no fragment-to-consensus mismatch rate exceeds 5%. (5) *Merging of reverse complements.* After bisulfite conversion, reads originating from the two strands of the same DNA fragment are often not identified as reverse complements during the Bowtie2 alignment and are therefore not automatically merged into one consensus. To overcome this problem, all reads that start and end with the RRBS restriction site (MspI: 5′ [CT]GG – [CT][CT]G 3′) are tested for whether they become perfect reverse complements of each other when all Cs are replaced by Ts and all Gs are replaced by As. For each pair to be merged, a consensus is formed by assigning a C to all T positions in the sequence of the forward partner at which the reverse-complement partner shows a C. (6) *Concatenation into one deduced genome.* In the final step, the merged deduced genome fragments are concatenated into one deduced genome that can be used for alignment, DNA methylation calling, and differential methylation analysis in the same way as a regular reference genome. To avoid creating artificial sequences at the concatenation sites, spacer sequences consisting of 50 Ns (equaling the read length) are added between the deduced genome fragments. Of note, all key parameters in RefFreeDMA have been empirically optimized and can be changed by the user of the software.

### Mapping and DNA Methylation Calling

Bisulfite alignment of the RRBS reads to the deduced genomes and to the reference genomes, as well as the mapping of the deduced genome fragments to the reference genomes was performed using BSMAP v2.74 (Xi and Li, 2009) with the following command line: *bsmap -a $input_fastq -d $ref_genome_fasta -o $output_bam -D C-CGG -w 100 -v 0.08 -r 1 -p 4 -n 0 -S 1 -f 5 –u*. For cross-mapping and alignment to the deduced genomes, the -D parameter was not set, disabling the RRBS mode to allow mapping of reads independently of restriction sites. Also, for cross-mapping, the maximum allowed error rate (*-v*) was set to 0.2. The human (hg19) and cow (bosTau6) reference genomes were downloaded from the UCSC Genome Browser, and the carp reference genome was downloaded from the European Nucleotide Archive (ENA) project PRJEB7241 assembly GCA_000951615.1. For better handling, the 9,377 scaffolds of the carp genome were concatenated into ten artificial chromosomes using stretches of Ns as separators. DNA methylation calling was performed using the *biseqMethCalling.py* software (Bock et al., 2010).

### Differential Methylation Analysis

CpG sites exhibiting differential DNA methylation between predefined groups of samples were identified using hierarchical linear models as implemented in the *limma* R package. Multiple testing correction was performed for CpG sites using the false discovery rate method implemented in R’s *p.adjust()* function. To assess the significance of differential DNA methylation for entire fragments, multiple testing corrected p values for all CpG sites contained in a fragment were combined using an extension of Fisher’s method (Makambi, 2003) as implemented in RnBeads (Assenov et al., 2014). Differentially methylated fragments were priority ranked based on statistical significance as well as effect size, calculating ranks individually for p value, log fold change, and absolute difference in DNA methylation levels and then selecting the worst of the three ranks as representative for the fragment. This way, fragments that achieve top ranks in all of the measures are favored, whereas fragments that are assigned a bad rank in one or more of the measures are penalized.

### Software Properties

RefFreeDMA is a Linux-based software pipeline that supports the various steps of reference genome independent analysis of differential DNA methylation based on RRBS data. External software requirements are limited to standard command line tools for next generation sequencing analysis, including picardtools, samtools, trimgalore, bowtie2, and bsmap. Runtime and memory usage depend on the number of samples, the number of reads per sample, the RRBS library complexity, and whether RefFreeDMA’s support for parallelization is used. For the presented datasets, which comprise 12 to 20 samples per species with ∼18 million 50-bp single-end reads per sample, one complete run using four cores (Intel Xeon E5-2650 processor) takes about 9 hr (wall-clock time) with parallelization and 40 hr (wall-clock time) without. The peak memory usage is 15 GB during consensus building. Although this study focuses on CpG methylation, our software also supports non-CpG methylation (when the *nonCpG* parameter is set to TRUE). RefFreeDMA is available as open source under the GPLv3 license: http://RefFreeDMA.computational-epigenetics.org.

### Comparison between Reference-Free and Reference-Based Analysis

Correspondence between the published reference genomes and the deduced genomes is determined by mapping the deduced genome fragments to the corresponding reference genome. The resulting associations between CpG sites in the deduced genome and the reference genome serve as the basis for the validations. Figure S2 depicts the correct match between the two approaches (case 1) as well as four scenarios in which discrepancies between reference-free and reference-based analysis are expected (cases 2 to 5). Comparisons between the reference-free and reference-based approaches are performed at the level of individual CpGs and at the level of deduced genome fragments.

### Cross-Mapping Analysis

In order to establish a connection between deduced genome fragments identified by RefFreeDMA in one species and well-annotated genomes of other species, deduced fragments were mapped to the human genome (hg19) and the mouse genome (mm10) using BSMAP/RRBSMAP with a maximum allowed mismatch rate of 20% as described in Mapping and DNA Methylation Calling. Overlaps between the genomic positions of mapped deduced genome fragments and annotations on the respective genome can then be used to perform enrichment analysis for the deduced fragments. We assessed differentially methylated fragments for enrichment of genomic annotations using LOLA (Sheffield and Bock, 2015). LOLA tests for significant enrichment of overlap between user-defined genomic regions of interest (i.e., the fragment mapping positions) and experimentally annotated genomic regions, which are provided as a database. The matched genomic regions for the differentially methylated fragments (mean coverage > 2 and adjusted p < 0.05) of granulocytes or lymphocytes were used as primary input regions (user set), while the genomic regions of all mapped deduced genome fragments were used as background (universe). The regions database for human (hg19) consisted of region sets downloaded from Cistrome, CODEX, ENCODE, and the UCSC Genome Browser as well as custom sets for DNase hypersensitivity sites (Sheffield et al., 2013). The region database for mouse (mm10) consisted of region sets downloaded from CODEX and ENCODE.

### Motif Enrichment Analysis

Motif enrichment analysis was performed using the command-line version of the AME tool (McLeay and Bailey, 2010) from the MEME package. We used the average odds score as sequence scoring method and the rank-sum test as motif enrichment test. All motifs were obtained from the JASPAR CORE (2014) Vertebrates database (Mathelier et al., 2014). Only enrichments with an adjusted p value lower than 0.05 were reported. In order to find motifs that are differentially enriched among differentially methylated fragments, the top-500 differentially methylated fragments (mean coverage > 2 and adjusted p < 0.05) of one sample group were used as primary input sequences, while the top-500 differentially methylated fragments of the other group were used as background (control sequences). To correct for motif enrichment due to base composition bias (Figures 5B and S5C), we performed the same analysis on random sequences that were constructed to reflect the base compositions of both groups on single nucleotide and dinucleotide level in 50 iterations each. To this end, the base compositions of the original sequences were determined using the fasta-get-markov tool from the MEME package. The 0^th^- and 1^st^-order Markov models for each group were then used as input for the gendb tool, which constructed 500 random sequences (length ∼50 bases) according to the models. This process was repeated 50 times with different random seeds. Finally, for each iteration AME was run on the shuffled sequences of one group as input and the shuffled sequences of the other group as background. All motifs that were detected as significantly enriched in more than 60% of all iterations were identified as false positives due to base composition bias and removed from the list of differentially enriched motifs identified for the original sequences. Furthermore, to identify motifs that might be enriched in differentially methylated fragments of both groups, we ran AME using the original sequences as input and the respective shuffled sequences as background. Only motifs that were found to be enriched in at least 95% of the iterations were reported as truly enriched in the differentially methylated fragments compared with the randomly shuffled sequences. For each enriched motif, the least significant p value was reported.

## Author Contributions

J.K. and C.B. designed the study. P.D., M.F., and C.B. optimized the RRBS protocol. J.K. acquired and prepared the samples. J.K., D.P., and G.F. performed FACS sorting. P.D. and J.H. made the RRBS sequencing libraries. J.K. developed RefFreeDMA and performed the computational analysis with input from N.C.S. and C.B. J.K. and C.B. wrote the manuscript with input from all co-authors.

## Accession Numbers

The DNA methylation data reported in this paper have been submitted to the NCBI GEO and are available under accession number GEO: GSE74026.

## Supplemental Information

Document S1. Figures S1–S5

Table S1. Summary Statistics for the Reference-Free and Reference-Based Analysis of DNA Methylation in the Blood Dataset, Related to Figure 2 For each of the analyzed samples and biological replicates, this shows the number of total reads, mapped reads, and informative reads (i.e., those that give rise to at least one valid DNA methylation measurement), mean DNA methylation levels of methylated and unmethylated spike-in controls, mean DNA methylation levels across CpG sites, non-CpG conversion rates, as well as the number of CpG measurements, number of covered CpGs, and mean informative sequencing coverage per CpG site.

Table S2. Summary Statistics for Direct Cross-Mapping of Carp RRBS Reads to the Human, Mouse, and Zebrafish Genome with Various Choices of Alignment Parameters, Related to Figure 5 For each of the carp samples, this lists the number of mapped reads, the percentage of mapped reads, and the number of CpGs covered using four different mapping approaches with different BSMAP parameters: maximum mismatch rate of 0.08 with multi-mapping reads, maximum mismatch rate of 0.08 without multi-mapping reads, maximum mismatch rate of 0.2 with multi-mapping reads, and maximum mismatch rate of 0.2 without multi-mapping reads.

Document S2. Article plus Supplemental Information
