# Supplementary material for: Differential DNA Methylation Analysis without a Reference Genome
Source: Cell Rep. 2015 Dec 8;13(11):2621–33. doi: 10.1016/j.celrep.2015.11.024 (PMC4695333; doi:10.1016/j.celrep.2015.11.024)
Supplement: Document S1. Figures S1–S5 [file mmc1.pdf]

Cell Reports

Supplemental Information

Differential DNA Methylation Analysis without a Reference Genome

Johanna Klughammer, Paul Datlinger, Dieter Printz, Nathan C. Sheffield, Matthias Farlik, Johanna Hadler, Gerhard Fritsch, and Christoph Bock

Supplemental Figures, Legends, and Tables

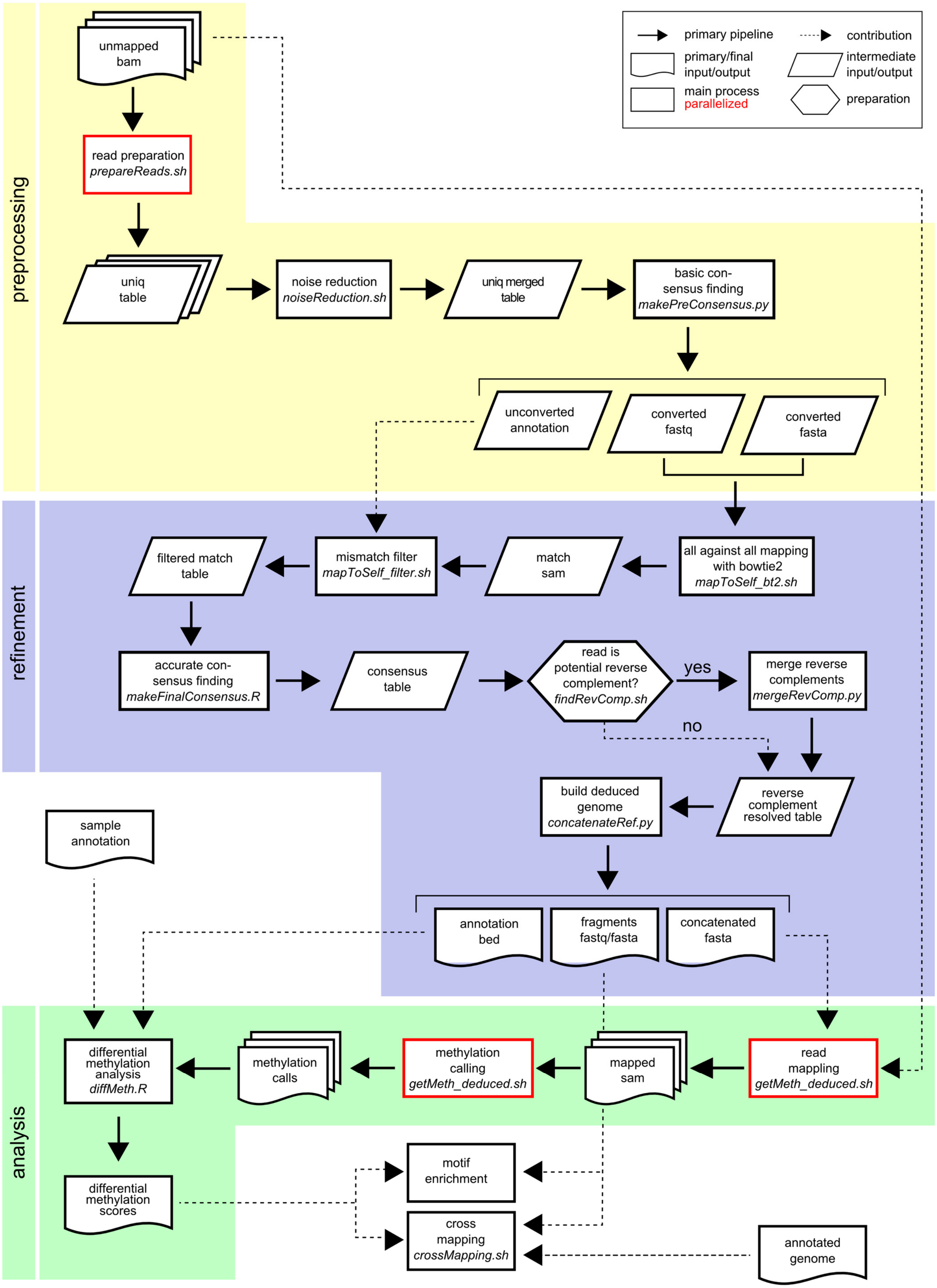

Figure S1. UML diagram outlining the RefFreeDMA software and analysis workflow, Related to Figure 1

The diagram illustrates the RefFreeDMA software and its key computational steps for performing reference-free analysis of differential DNA methylation, starting from raw RRBS reads and resulting in a ranked list of differentially methylated sites and fragments.

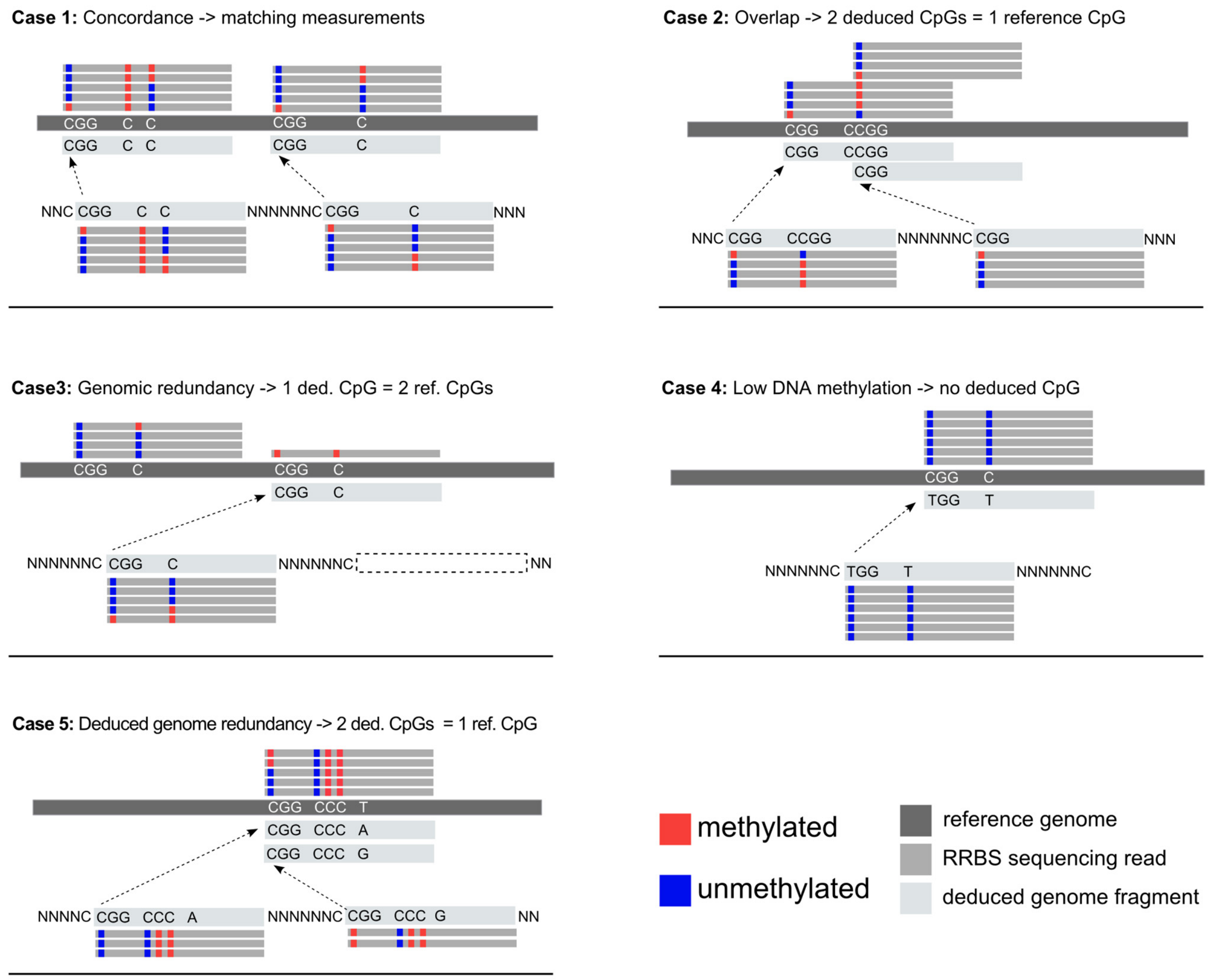

Figure S2. Sources of discrepancy between reference-free and reference-based analysis, Related to Figure 1

Case 1 depicts concordance between the two approaches, which applies to the vast majority of non-repetitive fragments that are not entirely unmethylated in all samples. All matching CpGs are uniquely assigned to each other when aligning the deduced genome fragments to the reference genome. *Case 2* depicts a scenario in which two deduced genome fragments overlap when aligned to the reference genome. Here, two measurements in the deduced genome are represented by only one measurement in the reference genome. *Case 3* depicts genomic redundancy caused by repetitive sequences in the reference genome. In the deduced genome, these similar or sequence-identical regions are represented by one deduced genome fragment. Multiple CpG sites in the reference genome are thus represented by only one site in the deduced genome. *Case 4* depicts the scenario where all reads are completely unmethylated for a given set of CpG sites. Deduced genome fragments covering these sites will contain a T instead of a C at the respective position, thereby reducing the number of CpG sites in the deduced genome. *Case 5* depicts the effect of deduced genome redundancy, which can occur when fragments contain sequencing errors that make them too dissimilar to be merged into one consensus.

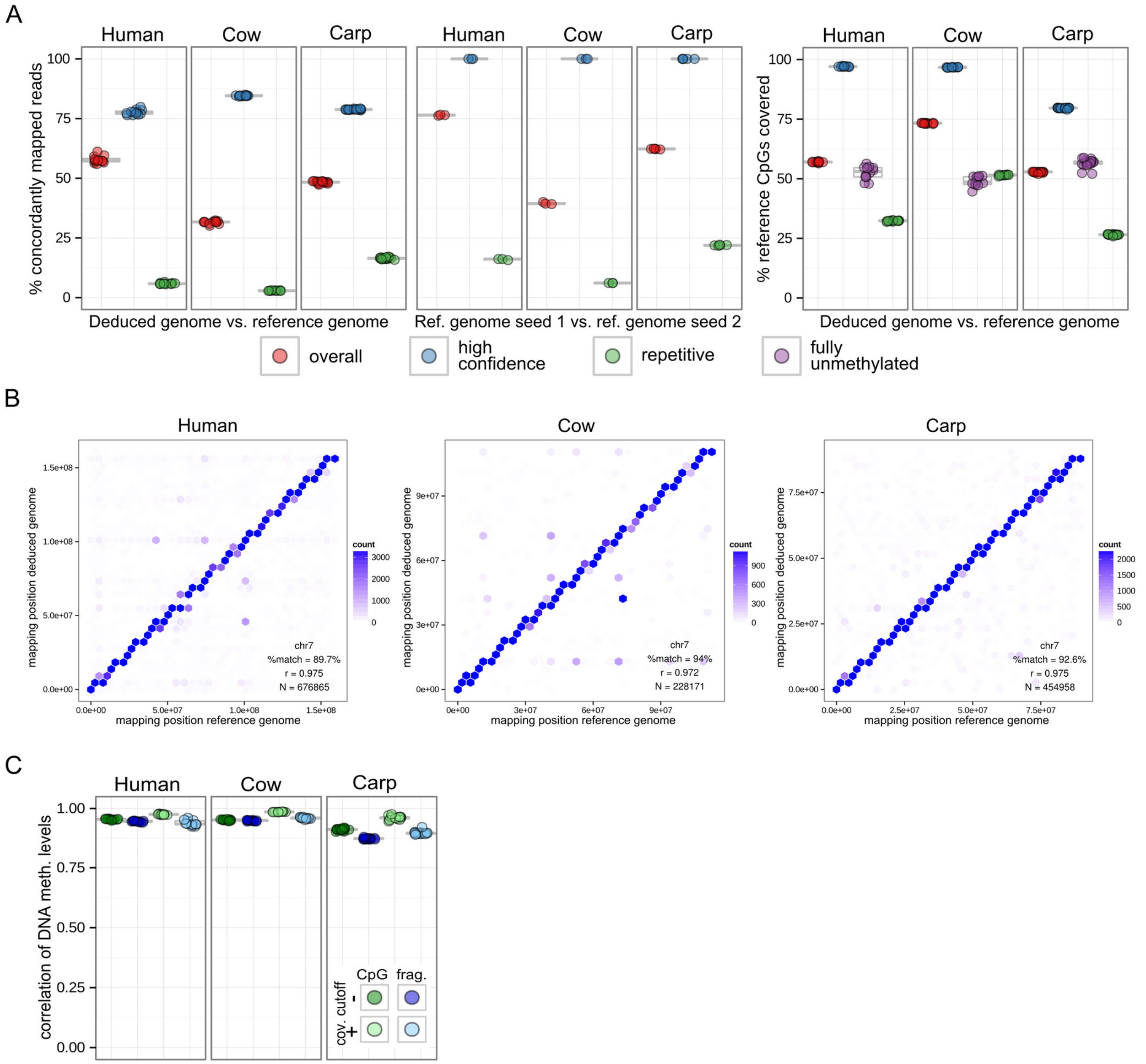

Figure S3. Comparison of reference-free & reference-based DNA methylation analysis, Related to Figure 3

(**A**) Concordance of mapped read positions (left) and covered CpG sites (right) between the reference-based and reference-free methods. For comparison, the concordance is also shown for the case of aligning the reads twice to the reference genome using different seeds for random assignment of reads that map to multiple positions (middle). "High confidence" fragments are those that are neither repetitive nor unmethylated in all samples. (**B**) Scatterplots illustrating the concordance of read mapping positions between the reference-free (y-axis) and reference-based (x-axis) methods. Representative plots of chromosome 7 are shown for each species (r: Pearson correlation; N: number of RRBS reads). (**C**) Pearson correlation of DNA methylation levels obtained with the two approaches, calculated for CpG sites as well as deduced genome fragments (frag.) with (+) and without (-) coverage filtering (requiring at least eight and not more than 200 mapped reads per CpG site or fragment).

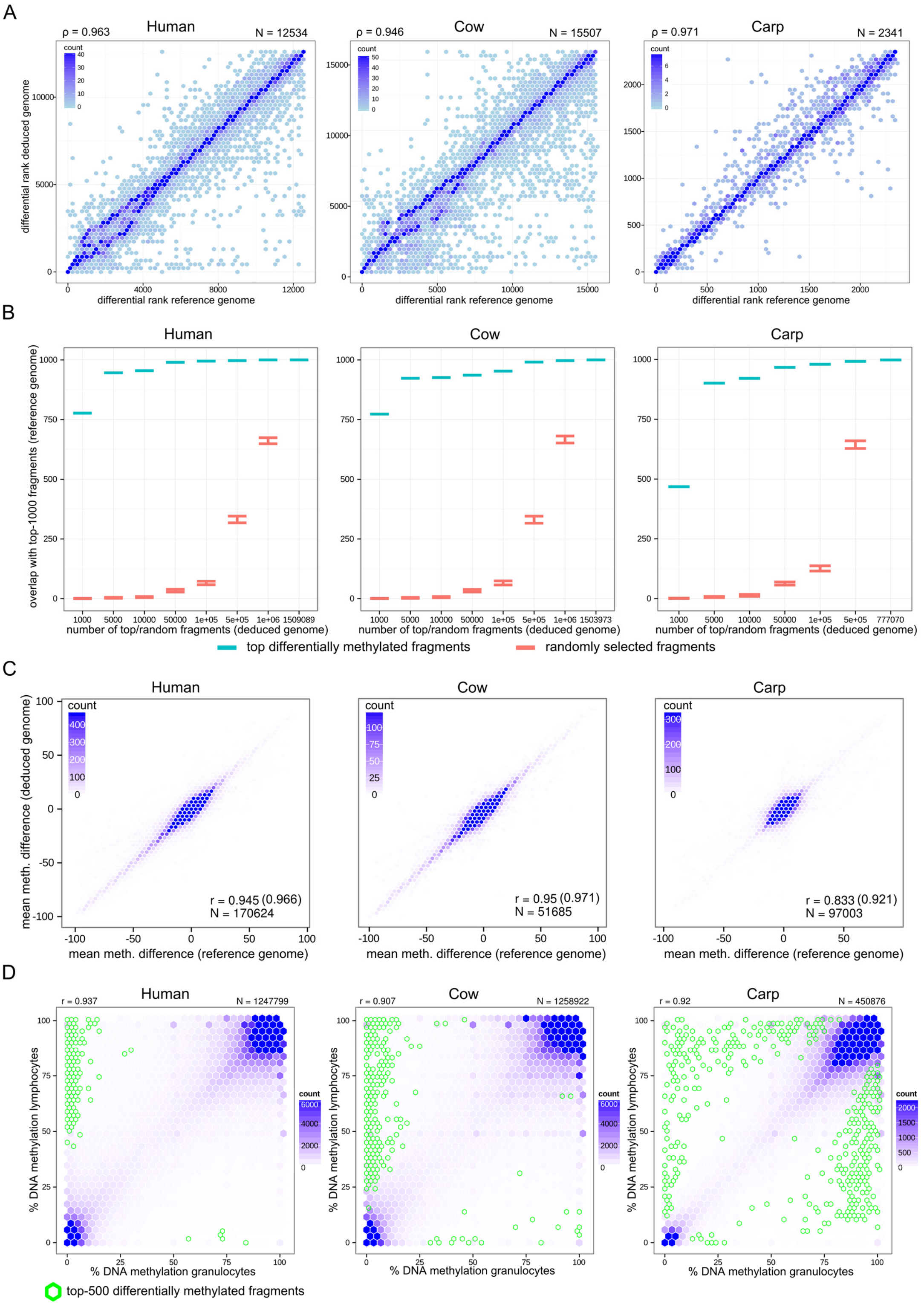

Figure S4. Validation of reference-free analysis of differential DNA methylation, Related to Figure 4

(**A**) Scatterplots displaying the agreement between differential methylation ranks for differentially methylated fragments (p-value < 0.05) using the two approaches (ρ: Spearman correlation coefficient; N: number of deduced genome fragments). (**B**) Recovery of the top-1000 differentially methylated deduced genome fragments (p-value < 0.05, coverage ≥ 8, non-overlapping) determined by the reference-based approach in a gradually increasing number of top differentially methylated deduced genome fragments using the reference-free approach (blue). The recovery within an equal number of randomly selected deduced genome fragments is shown for comparison (red). (**C**) Scatterplots showing the difference in mean fragment methylation between granulocytes and lymphocytes as determined by the reference-based (x-axis) vs. the reference-free (y-axis) approach for fragments that overlap with each other when mapped to the reference genome. Pearson correlations (r) for non-overlapping fragments are indicated in brackets. This plot shows that differential DNA methylation values are not strongly affected by overlapping fragments (Case 2 in Figure S2). All fragments were coverage-filtered for at least eight and not more than 200 mapped reads. (**D**) DNA methylation scatterplots demonstrating differential DNA methylation in granulocytes (x-axis) vs. lymphocytes (y-axis) using the reference-based approach. Means across four biological replicates are shown for each cell type, and the green hexagons indicate the top-500 most differentially methylated fragments. Matched scatterplots for the reference-free analysis are shown in Figure 4C.

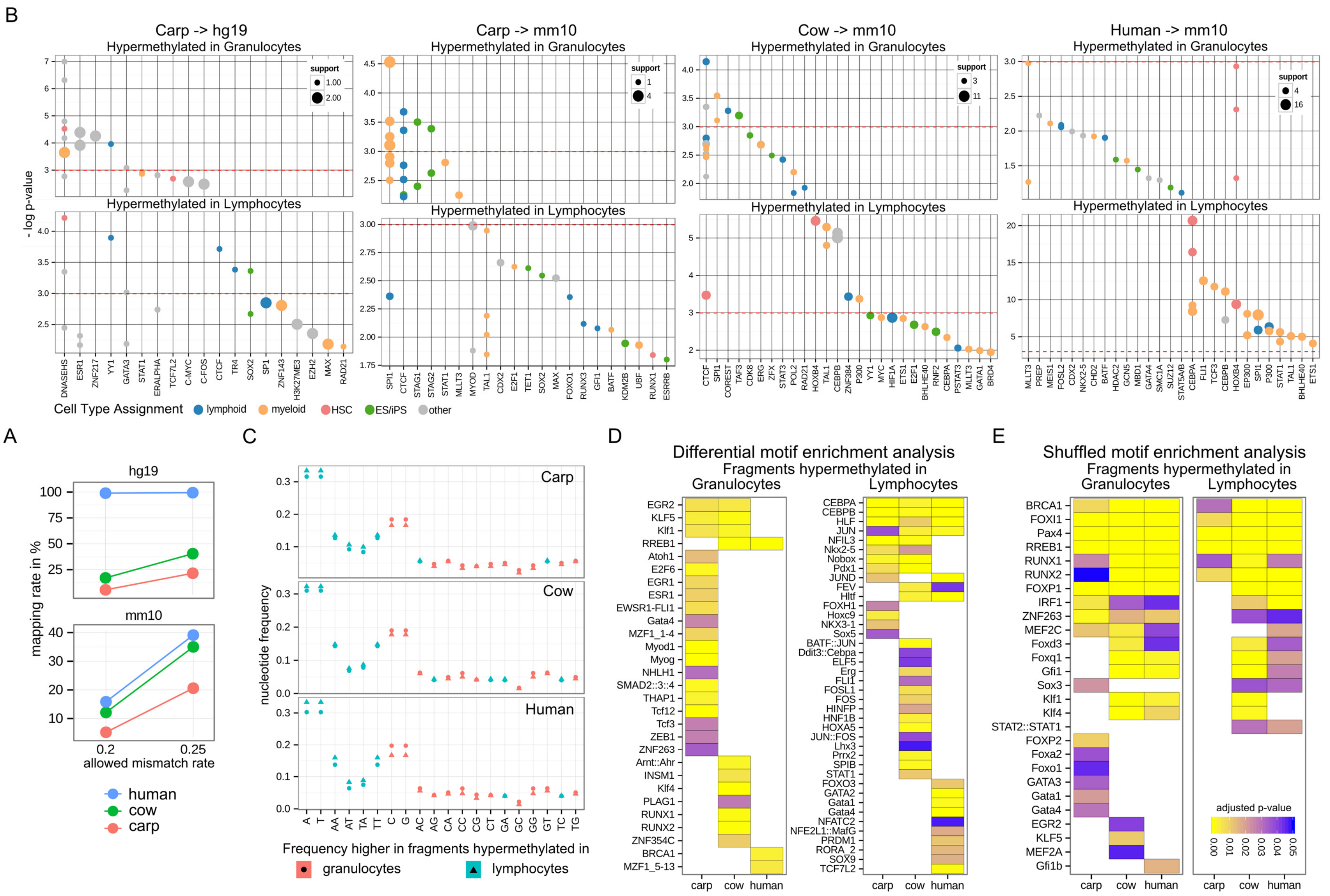

Figure S5: Interpretation of DNA methylation differences through cross-mapping to annotated genomes and motif enrichment analysis, Related to Figure 5

(**A**) Mapping of the deduced genome fragments of human, cow, and carp to the reference genomes of human (hg19) and mouse (mm10). Mapping rates are displayed for maximum mismatch rates of 20% and 25%. (**B**) Region enrichment analysis for reference-free deduced genome fragments that have been cross-mapped to the reference genomes of human (hg19) and mouse (mm10). For each group, the top-20 enrichments obtained by LOLA analysis are shown. Uncorrected p-values are plotted on the y-axis, and the number of overlapping regions is indicated by bubble size. Each dot represents an experiment listed in the database, and the red dashed lines indicate p-values of 0.05. Similar plots for human and cow cross-mapping to the human genome (hg19) are shown in Figure 5A. (**C**) Nucleotide frequency differences between the top-500 deduced genome fragments in granulocytes (dots) and lymphocytes (triangles). (**D**) Complete list of enriched sequence motifs from JASPAR CORE (2014) Vertebrates database among the top-500 deduced genome fragments with increased DNA methylation in granulocytes vs. lymphocytes (right) and vice versa (left). The motif analysis used the opposing group as background. (**E**) Same as in panel D, but using randomly shuffled sequences with the same mono- and dinucleotide composition as background. The displayed motifs were identified as significantly enriched in at least 95% of iterations.

Table S1. Summary statistics for the reference-free and reference-based analysis of DNA methylation in the blood dataset, Related to Figure 2

Table showing for each of the analyzed samples and biological replicates the number of total reads, mapped reads, and informative reads (i.e., those that give rise to at least one valid DNA methylation measurement), mean DNA methylation levels of methylated and unmethylated spike-in controls, mean DNA methylation levels across CpG sites, non-CpG conversion rates, as well as the number of CpG measurements, number of covered CpGs, and mean informative sequencing coverage per CpG site.

This table is provided as a separate Excel file.

Table S2. Summary statistics for direct cross-mapping of carp RRBS reads to the human, mouse, and zebrafish genome with various choices of alignment parameters, Related to Figure 5

Table listing for each of the carp samples the number of mapped reads, the percentage of mapped reads, and the number of CpGs covered using four different mapping approaches with different BSMAP parameters: Maximum mismatch rate of 0.08 with multi-mapping reads; maximum mismatch rate of 0.08 without multi-mapping reads; maximum mismatch rate of 0.2 with multi-mapping reads; and maximum mismatch rate of 0.2 without multi-mapping reads.

This table is provided as a separate Excel file
